# Bone mesenchymal stem cells-derived miR-223-3p-containing exosomes ameliorate lipopolysaccharide-induced acute uterine injury via interacting with endothelial progenitor cells

**DOI:** 10.1080/21655979.2021.2001185

**Published:** 2021-12-07

**Authors:** Yana Liu, Shihong Zhang, Zhiwei Xue, Xiaoxia Zhou, Lin Tong, Jiachen Liao, Huan Pan, Shu Zhou

**Affiliations:** a Department of Obstetrics and Gynecology, Key Laboratory of Obstetric and Gynecologic and Pediatric Disease and Birth Defects of Ministry of Education, West China Second University Hospital, Sichuan University, Chengdu, Sichaun, China; b Department of Obstetrics and Gynecology, Minerva Hospital for Women and Children, Chengdu, Sichuan, China; c Department of Obstetrics and Gynecology, Chengdu Second People’s Hospital, Chengdu, Sichuan, China

**Keywords:** Bone mesenchymal stem cells, endothelial progenitor cells, intrauterine adhesion, miR-223-3p, NLRP3-mediated pyroptotic cell death

## Abstract

Bone mesenchymal stem cells (BMSCs) have been used for the treatment of acute uterine injury (AUI)-induced intrauterine adhesion (IUA) via interacting with the endothelial progenitor cells (EPCs), and BMSCs-derived exosomes (BMSCs-exo) may be the key regulators for this process. However, the underlying mechanisms have not been studied. Based on the existed literatures, lipopolysaccharide (LPS) was used to induce AUI in mice models and EPCs to mimic the realistic pathogenesis of IUA *in vivo* and *in vitro*. Our data suggested that LPS induced apoptotic and pyroptotic cell death in mice uterine horn tissues and EPCs, and the clinical data supported that increased levels of pro-inflammatory cytokines IL-18 and IL-1β were also observed in IUA patients’ serum samples, and silencing of NLRP3 rescued cell viability in LPS-treated EPCs. Next, the LPS-treated EPCs were respectively co-cultured with BMSCs in the Transwell system and BMSCs-exo, and the results hinted that both BMSCs and BMSCs-exo reversed the promoting effects of LPS treatment-induced cell death in EPCs. Then, we screened out miR-223-3p, as the upstream regulator for NLRP3, was enriched in BMSCs-exo, and BMSCs-exo inactivated NLRP3-mediated cell pyroptosis in EPCs via delivering miR-223-3p. Interestingly, upregulation of miR-223-3p attenuated LPS-induced cell death in EPCs. Collectively, we concluded that BMSCs-exo upregulated miR-223-3p to degrade NLRP3 in EPCs, which further reversed the cytotoxic effects of LPS treatment on EPCs to ameliorate LPS-induced AUI.

## 1. Introduction

To our knowledge, the main causes of intrauterine adhesion (IUA) in clinic are intrauterine surgery caused uterine damages [1,2] and infection [3], which subsequently lead to chronic inflammation and aggravate the progression of IUA [1–3]. IUA particularly occurs in women after pregnancy with low-levels of estradiol [4,5], and brings huge health burdens for women worldwide, including infertility [6], menstrual irregularities [7], and so on. Unfortunately, up until now, the effective treatment strategies for IUA in clinic have not been developed due to the complicated pathogenesis of IUA [8,9]. Endothelial progenitor cells (EPCs), also known as angioblast, are motivated from bone marrow to peripheral blood to repair the blood vessels injury via differentiating into vascular endothelial cells under physiological or pathological stimulations [10,11]. According to the existed information that EPCs mediated angiogenesis is pivotal for IUA recovery [12,13], this study selected primary EPCs for further investigations. Lipopolysaccharide (LPS) is the main pathogen of gram-negative bacteria [14,15], and according to the existed literature [16], LPS can be used to induce the cellular and animal models for acute uterine injury (AUI) and IUA, and the biological influences of LPS treatment on EPCs have been investigated [17,18].

Data from different research groups suggest that bone mesenchymal stem cells (BMSCs) can be employed to treat various diseases, such as kidney associated diseases [19,20], liver associated diseases [21–23], and IUA [24–26]. Specifically, Yao et al. report that BMSCs-derived exosomes (BMSCs-exo) contribute to the repair of damaged endometrium by regulating the TGF-β1/Smad pathway [26], and Tan et al. find that BMSCs-exo suppress fibrosis during endometrial repair of IUA [25]. Interestingly, Yu et al. report that BMSCs interact with endothelial progenitor cells (EPCs) to induce endometrium angiogenesis by modulating the PI3K/Akt/Cox2 signal pathway, which indirectly promotes the recovery of IUA [24], however, the detailed underlying mechanisms of BMSCs-EPCs interactions are still unclear. In addition, chronic or acute inflammation induced endometrial injury is decisive to accelerate the development of IUA [3], and NLRP3-mediated pyroptotic cell death is defined as a type of pro-inflammatory type of cell death [21–23], which contributes to LPS-induced endometritis and uterine injury/inflammation [27]. As previously reported [28,29], the expression levels of NLRP3, ASC and N-terminal Gasdermin D are commonly used as pivotal indicators for cell pyroptosis, thus we selected those biomarkers in our experiments.

It has been commonly reported that BMSCs interact with other type of cells through secreting exosomes to modulate the development of those inflammation-associated diseases [21,30,31], and those BMSCs-exo are enriched with microRNAs (miRNAs) [32–34]. MiRNAs are a class of small non-coding RNAs which regulate their downstream target genes by directly binding to the 3ʹ untranslated regions (3ʹUTR) of those genes [35,36], and various miRNAs that have been identified to be closely associated with IUA development [37,38]. In addition, BMSCs also secret miRNAs-containing exosomes to modulate IUA progression, for example, BMSCs-derived miR-29a containing exosomes promote IUA repair [25]. Among all the miRNAs, previous data show that miR-223-3p is enriched in BMSCs-exo, and BMSCs secret miR-223-3p-containing exosomes for liver protection in the animals with experimental autoimmune hepatitis [21]. Interestingly, miR-223-3p is able to target the 3ʹUTR of NLRP3 mRNA for its degradation and inhibition, resulting in the inhibition of NLRP3-mediated cell pyroptosis [39–41]. Moreover, miR-223-3p targets NLRP3 to attenuate inflammation in thromboangiitis obliterans rats and acute gouty arthritis [42,43].

Thus, this study aimed to explore the underlying mechanisms by which BMSCs exerted their curative effects on IUA. To achieve this, LPS was used to establish IUA models *in vitro* and *in vivo*, which were subsequently co-cultured with BMSCs or BMSCs-exo. The gain-of-function experiments were conducted, and the indicators for apoptotic and pyroptotic cell death were evaluated.

## 2. Materials and methods

### 2.1. LPS-induced IUA models in mice models

LPS was used to induce IUA models in female BALB/c mice (N = 6, 8–14 weeks) in accordance with the existed experimental protocols. The BALB/c mice were obtained from Research Animal Center of Sichuan University, and were housed in the conditions with water and food ad libitum, and the mice experienced 12 h light-dark cycle. LPS (0.5 mg/kg, Sigma, USA) was peritoneally injected to the BALB/c mice for 6 h to induce endometrial injury, and the mice were equally grouped into two: Control (injection with same volume of PBS), and LPS treatment group. At 12 h post-injection, mice were sacrificed and the uterine horn tissues were collected by cervical dislocation for further analysis. Also, the mice serum samples were collected and stored at −70 °C refrigerator for further utilization. The associated animal experiments were approved by the Ethics Committee of Sichuan University.

### 2.2. Isolation, purification and culture of BMSCs and EPCs in vitro

BMSCs were directly purchased from American Type Culture Collection (ATCC, #PCS-500-012^TM^, USA) and were cultured in the DMEM medium (Gibco, USA) following the ATCC’s instructions under the conditions with 5% CO_2_ atmosphere and 37 °C temperature. In addition, the EPCs were isolated and purified from human peripheral blood by using a Ficoll-Paque PLUS reagent (GE, USA) according to the previous work, which were subsequently cultured *in vitro* in the endothelial cell medium for 6 days. Then, cells were incubated with antibodies against CD34, CD133, and CD29, and the CD34^+^ CD133^+^CD29^+^ EPCs were obtained by using the Flow Cytometer (BD Bioscience, USA). The EPCs were subjected to 50 μg/ml LPS to establish LPS-induced cell injury models in EPCs.

### 2.3. Vectors transfection

The NLRP3 knock-down vectors, miR-223-3p mimic and inhibitor were designed according to the sequences provided by the previous publications [39,44], and were synthesized by a commercial third-party company (Sangon Biotech, China). Then, the NLRP3 silencing vectors (150 nM), miR-223-3p mimic (200 nM) and inhibitor (100 nM) were delivered into the EPCs for genes manipulation by using the Lipofectamine 2000 reagent (Invitrogen, USA) in keeping with the producer’s protocols and previous literatures [39,44]. At 48 h post-transfection, the transfection efficiency of the above vectors was examined by using the Real-Time qPCR analysis.

### 2.4. Establishment of the BMSCs-EPCs co-culture system

A Transwell system was used to establish the BMSCs-EPCs co-culture system according to the previous literatures. The Transwell chambers were purchased from Corning (USA), and the BMSCs and EPCs were respectively cultured in the lower and upper chamber of the transwell system at the ratio of 2:1. Then, those two cells were cultured in the system for 48 h at the standard culture conditions with 5% CO_2_ at 37 °C, and the EPCs were obtained for further analysis.

### 2.5. Real-Time qPCR analysis

The total RNA was extracted from EPCs by using the TRIzol reagent (Takara, Japan), and the RNA quality was examined by using the agarose electrophoresis. Then, the cDNA was obtained by conducting reverse transcription experiments through using the Reverse Transcription kit (Takara, Japan). The SYBR Premix Ex Taq^TM^ kit (Takara, Japan) was used to amplify the cDNA samples, which were subsequently analyzed by using the Applied Biosystems 7500 system (ThermoFisher Scientific, USA). The primer sequences for IL-1β, IL-18, miR-223-3p, NLRP3, GAPDH, U6, Bax and Bcl-2 could be found in the previous publications [39,44–46]. miR-223-3p expressions were normalized by U6, and other genes were normalized by GAPDH.

### 2.6. Western blot analysis

Total proteins were obtained from tissues and cells by using the RIPA lysis buffer (Sigma-Aldrich, USA), which were separated by using the 10% SDS-PAGE assay. The proteins in the gels were transferred to PVDF membranes (Millipore, USA), which were incubated with 5% nonfat milk for incubation for 1 h at room temperature. Next, the PVDF membranes were incubated with the primary antibodies against NLRP3 (1:1500, Catalog MAB7578, R&D system, USA), ASC (1:1000, Catalog AF3805, R&D system, USA), N-Gasdermin D (1:1000, Catalog ab209845, Abcam, UK), GAPDH (1:2000, Catalog 2275-PC-100, R&D system, USA), cleaved Caspase-3 (1:1500, Catalog AF835, R&D system, USA), Bax (1:2000, AF820, R&D system, USA), Cyclin D1 (1:1500, Catalog MAB4314, R&D system, USA) and CDK2 (1:2000, Catalog AF4654, R&D system, USA) at 4 °C overnight. Then, the membranes were incubated with the horseradish peroxidase-conjugated goat-anti-rabbit secondary antibody (Invitrogen, USA), and the protein bands were visualized by using the ECL kit (GE Healthcare, USA) and gray values were quantified by the Image J software.

### 2.7. Measurement of relative luciferase activities

The binding sites in miR-223-3p and 3ʹUTR of NLRP3 mRNA were obtained from the existed literatures [39,44], and following dual-luciferase reporter gene system assay was performed to validate those sites. Specifically, those sites in NLRP3 were mutated, and named as Mut-NLRP3. Correspondingly, the wild-type NLRP3 was indicated by using the term Wt-NLRP3. The above sequences were cloned into the luciferase reporter vectors, which were co-transfected with miR-223-3p mimic into the HEK-293 T cells. At 24 h post-transfection, a dual-luciferase reporter assay system (Promega, USA) was employed to detect relative luciferase activities.

### 2.8. Measurement of pro-inflammatory cytokines secretion

The expression levels of pro-inflammatory cytokines in the EPCs’ supernatants and mice serum, including IL-1β, IL-18, TNF-α and IL-4, were measured by using the corresponding ELISA kits (Beyotime, China) in keeping with the manufacturer’s protocol. The optical density (OD) values were measured to represent the relative expression levels of the above cytokines.

### 2.9. MTT assay for cell viability

The EPCs cells were obtained and cultured in the 96-well plates with the density of 3,000 cells per well, and were exposed to 50 μg/ml LPS for 0 h, 24 h, 48 h and 72 h, respectively. The cells were incubated with 20 μl MTT solution for 4 h at 37 °C, and the supernatants in each well were removed and discarded. Then, the cells were mixed with 150 μl DMSO to resolve formazan, and the 96-well plates were fully vortexed and the optical density (OD) values in each well were examined by using the microplate reader (ThermoFisher, USA), and the OD values could be used to represent relative cell viability.

### 2.10. Examination of cell apoptosis

The EPCs cells were exposed to 50 μg/ml LPS for 0 h and 24 h, and cell apoptosis ratio was determined by using the Apoptosis Detection kit (ThermoFisher, USA). The EPCs cells were fixed with 4% paraformaldehyde for 15 min at 4 °C, and were respectively stained with Annexin V-FITC and PI for 30 min at room temperature without light exposure. Then, a flow cytometer (BD Biosciences, USA) was employed to examine the Annexin V-FITC/PI-positive apoptotic cells among the EPCs cells, and the results were analyzed by using the FlowJo 7.2 software.

### 2.11. Purification of BMSCs-exo

We isolated the BMSCs-exo according to the experimental protocols documented in the previous literature [24]. Briefly, BMSCs were cultured under the standard culture conditions, and the supernatants were collected and filtered by a filtering membrane with 0.22 nm. The supernatants were then experienced the centrifugation procedures as follows: 2,000 g/20 min; 16,500 g/45 min; 100,000 g/2 h; and 100,000 g/ 2 h, and the temperature for centrifugation was set at 4 °C. After all the above procedures, the BMSCs-exo were obtained by using the commercial Exosome Isolation kit (Invitrogen, USA) according to their protocols.

### 2.12. Immunofluorescence staining assay

The EPCs cells were co-cultured with BMSCs-exo for 24 h, and the EPCs were isolated for further analysis. The cells were fixed by 4% formaldehyde (20 min, room temperature), and were permeabilized by 0.1% Triton X-100 (10 min, room temperature). Then, the EPCs cells were stained with fluorescent-labeled PKH67 for the BMSCs-exo and Hoechst for the nucleus of EPCs, and the merged images were generated to observe the process that BMSCs-exo incorporated into the EPCs by performing the confocal microscopy observations. The BMSCs-exo were stained with green, and the EPCs cells’ nucleus were stained in blue.

### 2.13 Data collection and analysis

The SPSS 22.0 software was used for data analysis. The data was represented as Means ± Standard Deviations (SD), and two-tailed unpaired Student’s t-test was performed to compare the means from two groups, and one-way ANOVA analysis was conducted for the comparisons of the means from multiple groups, which were corrected by using the Bonferroni method. *P* < 0.05 was regarded as statistical significance, and the ‘*’ symbol was used to indicate the significant differences.

## 3. Results

### 3.1. Establishment of in vitro and in vivo models for IUA by using LPS stimulations

**Figure 1.**
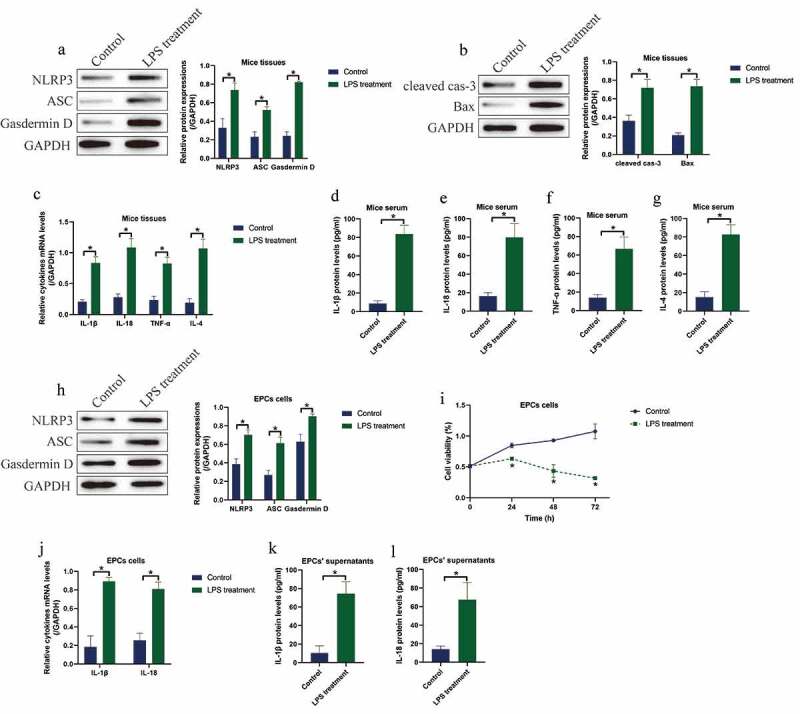
LPS induced cell pyroptosis, apoptosis and pro-inflammatory cytokines secretion in both IUA mice and cellular models. The expression status of (a) NLRP3, ASC and N-Gasdermin D, and (b) cleaved caspase-3 and Bax were detected by performing Western blot analysis. The pro-inflammatory cytokines generation and secretion were examined by (c) Real-Time qPCR in EPCs cells and (d-g) its supernatants were examined by Real-Time qPCR and ELISA assay. (h) Western blot was used to determine cell pytoptosis in EPCs cells. (i) MTT assay was used to determine cell viability in EPCs cells. (j) Generation and (k, l) secretion of IL-1β and IL-18 were respectively examined by Real-Time qPCR and ELISA. Individual experiment repeated 3 times, and *P < 0.05

Initially, we investigated the involvement of pyroptotic cell death in regulating LPS-induced IUA pathogenesis. To investigate this issue, we established the animal and cellular models for IUA by using the LPS treatment method according to the previous literature [16]. The BALB/c mice were intraperitoneally injected with LPS (0.7 mg/kg) for 24 h to induce *in vivo* IUA models, and the mice uterine horn tissues were collected. As shown in Figure 1(a,b), LPS significantly upregulated the expression levels of NLRP3, ASC and N-Gasdermin D to promote cell pyroptosis (Figure 1(a)), and promoted the expression levels of cleaved Caspase-3 and Bax (Figure 1(b)) and the ratio of Bax/Bcl-2 (Figure S1) in mice tissues. Consistently, the expression levels of the pro-inflammatory cytokines (IL-1β, IL-18, TNF-α and IL-4) were upregulated by LPS treatment at both transcriptional (Figure 1(c)) and translated (Figure 1(d,G)) levels in mice tissues and serum. In addition, the EPCs were isolated and cultured *in vitro*, which were subsequently stimulated with LPS (30 μg/ml) for 6 h to establish cellular IUA models. The results showed that LPS induced pyroptotic cell death (Figure 1(h)) and suppressed cell viability (Figure 1(i)) in EPCs. Moreover, the Real-Time qPCR (Figure 1(j)) and ELISA (Figure 1(k,l)) analysis validated that LPS promoted IL-1β and IL-18 generation and secretion in EPCs and its supernatants.

### 3.2. Silencing of NLRP3 rescued cell viability in LPS treated EPCs

**Figure 2.**
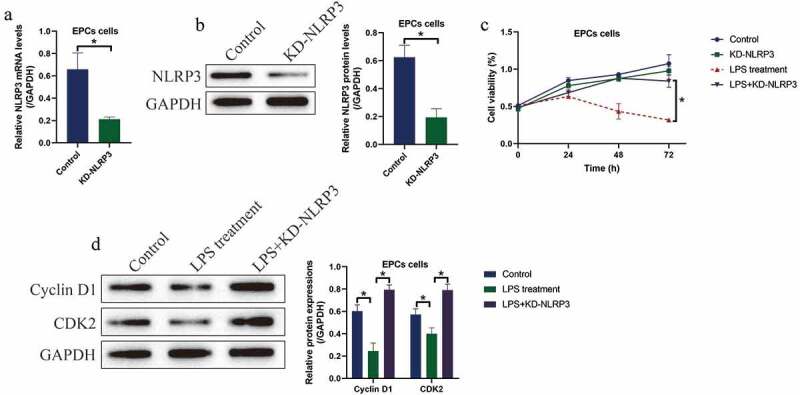
LPS suppressed EPCs cell viability via inducing cell pyroptosis. (a, b) NLRP3 was silenced in EPCs cells. (c) MTT assay was used to examine cell viability. (d) The expression status of Cyclin D1 and CDK2 were determined by Western blot analysis. Individual experiment repeated 3 times, and *P < 0.05

Then, we investigated whether LPS induced cell death in EPCs via triggering NLRP3-mediated pyroptotic cell death, and the NLRP3 knockdown vectors were successfully delivered into the EPCs (Figure 2(a,b)). We divided the cells into four groups, including Control, NLRP3 ablation alone group, LPS treatment group, and LPS+NLRP3 knockdown group. Analysis of the MTT data showed that knock-down of NLRP3 had little effects on EPCs viability, while NLRP3 ablation rescued cell viability in LPS-treated EPCs (Figure 2(c)), hinting that LPS promoted cell death in EPCs in a NLRP3-dependent manner. Similarly, the expression levels of the cell-cycle associated proteins, including Cyclin D1 and CDK2, were examined by Western blot, and we found that the inhibiting effects of LPS treatment on the above proteins were abrogated by co-transfecting cells with silencing vectors for NLRP3 (Figure 2(d)).

### 3.3. BMSCs and BMSCs-exo exerted protective effects on LPS-induced cell death in EPCs

**Figure 3.**
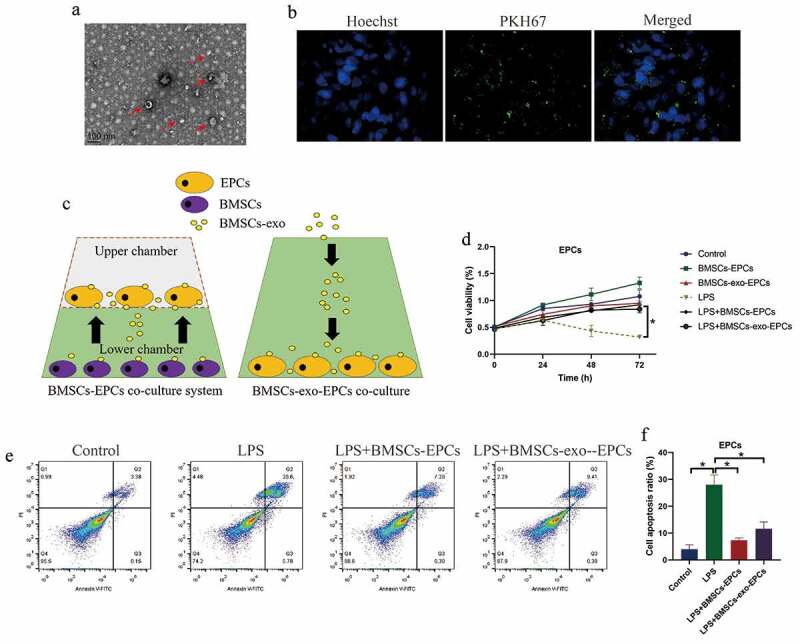
BMSCs restored cell viability in LPS-treated EPCs cells by secreting exosomes. (a) BMSCs-exo was observed and photographed by EM, and (b) were identified by fluorescence staining assay. (c) The schematic design for BMSCs-EPCs and BMSCs-exo-EPCs co-culturing system. (d) Cell viability and (e, f) apoptosis were respectively determined by MTT assay and FCM assay. Individual experiment repeated 3 times, and *P < 0.05

Based on the previous experimental protocols [24], the BMSCs and BMSCs-exo were respectively isolated and prepared. The BMSCs-exo was observed under transmission electron microscope (TEM) (Figure 3(a)), and identified by staining with PKH67 protein (Figure 3(b)). The BMSCs were co-cultured with the EPCs in a Transwell system to avoid direct cell-to-cell contact, and the BMSCs-exo (10 μg exosomes per 100 μl) was directly incubated with EPCs in the 96-well plates (Figure 3(c)). Next, the EPCs were stimulated with LPS, and were divided into six groups, including Control, LPS treatment, BMSCs-EPCs co-culture, BMSC-exo-EPCs co-culture, LPS+EPCs (BMSCs co-culture), and LPS+EPCs (BMSCs-exo co-culture), and the EPCs were obtained for further analysis. As shown in Figure 3(d), both BMSCs and BMSCs-exo slightly increased cell proliferation abilities in EPCs, in contrast with the EPCs alone group. Also, the EPCs were stained with Annexin V-FITC and PI, and FCM was performed to examine the Annexin V-FITC/PI-positive apoptotic cells. As expected, our data showed that LPS-induced cell apoptosis in EPCs were reversed by co-treating cells with both BMSCs and BMSCs-exo (Figure 3(e,f)).

### 3.4. BMSCs-exo delivered miR-223-3p to inactivate LPS-induced pyroptotic cell death in EPCs

**Figure 4.**
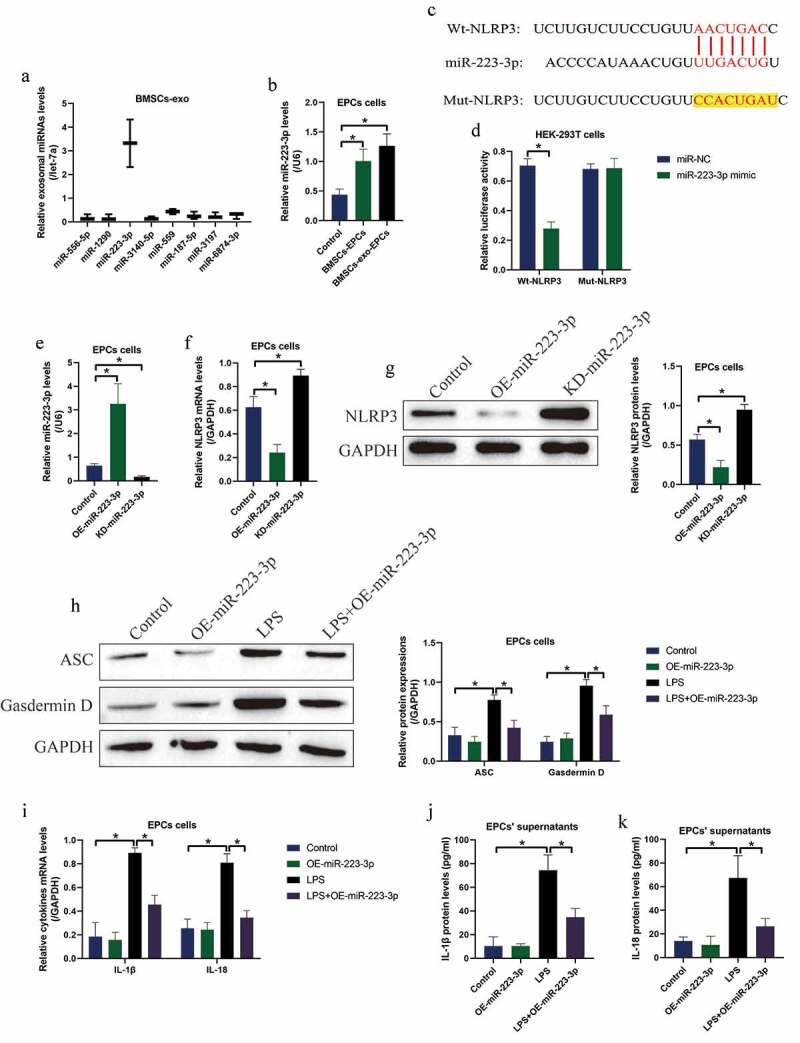
MiR-223-3p was capable of regulating NLRP3-mediated pyroptotic cell death in EPCs. (a) The eight miRNAs in BMSCs-exo were screened by using Real-Time qPCR, and (b) the process that BMSCs-exo delivered miR-223-3p into EPCs was examined. (c) The targeting sites in miR-223-3p and 3ʹ UTR of NLRP3 mRNA were predicted, and (d) those sites were validated by performing dual-luciferase reporter gene system assay. (e) miR-223-3p was overexpressed and silenced, and (f, g) the following experiments validated that miR-223-3p negatively regulated NLRP3 in EPCs cells. Upregulation of miR-223-3p suppressed LPS-induced (h) pyroptotic cell death, (i) cytokines generation and (j, k) secretion in EPCs cells. Individual experiment repeated 3 times, and *P < 0.05

Previous literatures reported that BMSCs interact with other cells via secreting miRNAs-containing exosomes [21,30,31], and NLRP3 can be targeted by various miRNAs [39–41], which encouraged us to investigate whether BMSCs or BMSCs-exo delivered exosomal miRNAs to rescue cellular functions in the LPS-treated EPCs. Hence, we screened the upstream eight miRNAs that targeted NLRP3, and found that miR-223-3p, instead of other miRNAs, was enriched in the BMSCs-exo, which was normalized by let-7a miRNA (Figure 4(a)). Also, co-culture of both BMSCs and BMSCs-exo with EPCs was capable of increasing the expression levels of miR-223-3p in the EPCs (Figure 4(b)). Moreover, the biding sites of miR-223-3p with NLRP3 mRNA were predicted (Figure 4(c)), which were validated by performing the following dual-luciferase reporter gene system assay (Figure 4(d)). Then, miR-22-3p was respectively silenced and overexpressed in the EPCs cells (Figure 4(e)), and the following assay evidenced that miR-223-3p targeted NLRP3 for its suppression and inhibition (Figure 4(f,g)). Finally, overexpression of miR-223-3p also decreased the expression levels of ASC and N-Gasdermin D to restrain LPS-induced cell pyroptosis in EPCs (Figure 4(h)), and LPS-induced IL-1β and IL-18 generation (Figure 4(i)) and secretion (Figure 4(j,k)) was also hampered by miR-223-3p upregulation in the EPCs-derived supernatants.

### 3.5. Overexpression of miR-223-3p attenuated LPS-induced cell death in EPCs

**Figure 5.**
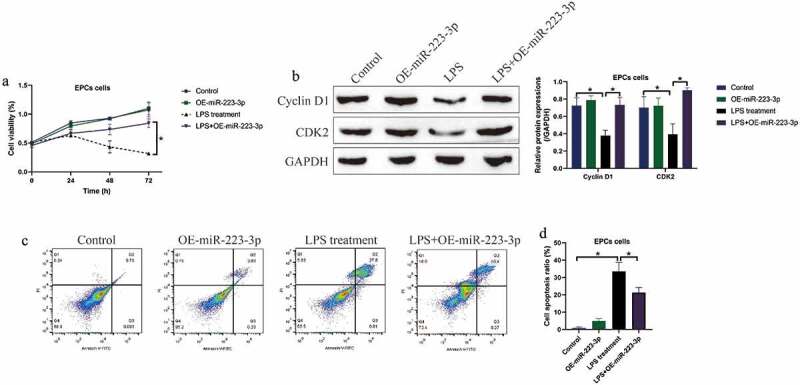
Upregulated miR-223-3p reversed LPS-induced cell death in EPCs cells. (a) MTT assay was conducted to determine cell viability at different time points. (b) The cell cycle-associated proteins were measured by performing Western blot analysis. (c, d) FCM assay was used to examine cell apoptosis ratio in EPCs cells. Individual experiment repeated 3 times, and *P < 0.05

Given that miR-223-3p acted as a post-transcriptional regulator for NLRP3, and silencing of NLRP3 rescued cell viability in EPCs with LPS stimulation. We next explored whether miR-223-3p was effective to restore cell viability in LPS treated EPCs. The MTT assay results in Figure 5(a) suggested that upregulation of miR-223-3p significantly increased cell viability in EPCs treated with LPS, and further Western blot analysis results supported that miR-223-3p overexpression increased the expression levels of Cyclin D1 and CDK2 in LPS treated EPCs (Figure 5(b)). Consistently, we performed FCM assay to detect cell apoptosis, and the results showed that overexpression of miR-223-3p also restrained LPS-induced apoptotic cell death in EPCs (Figure 5(c,d)).

## 4. Discussion

Acute uterine injury (AUI)-induced intrauterine adhesion (IUA) seriously degrades the life quality of women worldwide [6,7], and our knowledge in the molecular mechanisms of IUA pathogenesis is scarce, which hampers the development of effective treatment strategies for IUA in clinic [8,9]. Hence, it is urgent for the researchers to solve this issue by conducting fundamental experiments. To our knowledge, induction of angiogenesis improves IUA recovery [12,13], and according to the existed information, endothelial progenitor cells (EPCs) are pivotal for promoting angiogenesis via differentiating into vascular endothelial cells under physiological or pathological stimulations [10,11], which also contribute to the recovery of AUI and IUA [24]. Nevertheless, suppression of EPCs by environmental stress, such as bacterial infection and inflammation [1–3], makes this recovering process invalid. To investigate this issue, we used lipopolysaccharide (LPS) stimulation to establish IUA models in both mice and EPCs [16], which simulated the realistic conditions of gram-negative bacterial infection. As expected, our data supported that LPS induced both apoptotic and pyroptotic cell death in the IUA models, and the inhibiting effects of LPS on EPCs viability were abrogated by knocking down NLRP3. Our results were supported by the previous literatures that LPS-induced both cell pyroptosis [47] and apoptosis [48].

Bone mesenchymal stem cells (BMSCs) implantation has been proved as an effective strategy to treat various diseases [21,30,31], which also participate in the regulation of IUA progression [24–26]. As previously described, BMSCs commonly exert their biological functions through secreting exosomes [21,30,31], which contain proteins or non-coding RNAs that are crucial for regulating cellular functions [32–34]. Of note, Yu et al. report that interaction between BMSCs and EPCs facilitate EPCs-induced angiogenesis in a transwell system that avoids direct cell-to-cell contact [24], but the authors do not explore by which means BMSCs interact with EPCs. Based on this, we respectively co-cultured the LPS-treated EPCs with BMSCs in a transwell system and BMSCs-derived exosomes (BMSCs-exo), and our data supported the published data that BMSCs rescued cell viability in LPS-treated EPCs via secreting BMSCs-exo [21,30,31].

As previously described, there exist various contents in the BMSCs-exo that exert their biological functions, and microRNAs (miRNAs) have been identified as one of the most important contents in the BMSCs-exo [32–34]. In addition, miRNAs exert their biological functions through serving as post-transcriptional regulators to target the 3ʹUTR of their downstream targets [35,36], and NLRP3 can be targeted via multiple miRNAs [37,38]. The above information encouraged us to investigate whether BMSCs-exo protect EPCs from LPS-induced cell death via delivering NLRP3’s upstream miRNAs. Thus, we screened the expression status of miRNAs that targeted NLRP3 in the BMSCs-exo, and surprisingly found that miR-223-3p, instead of other miRNAs, was enriched in the BMSCs-exo, and previous publications supported our conclusion that BMSCs secreted miR-223-3p-containing exosomes [21]. In addition, we evidenced that BMSCs-exo delivered miR-223-3p to EPCs for its upregulation, which subsequently suppressed LPS-induced cell pyroptosis by degrading NLRP3 in a ceRNA-dependent manner, which were in consistence with the previous work [42,43].

## 5. Conclusions

We concluded that BMSCs delivered exosomal miR-223-3p to EPCs to degrade NLRP3 in a ceRNA mechanisms-dependent manner, resulting in the inhibition of LPS-induced cell death and inflammation in EPCs, which promoted EPCs-mediated angiogenesis and IUA recovery. However, since we draw the above conclusions mainly from our *in vitro* experiments, and future *in vivo* validation experiments were still needed to support our current conclusions.

## Supplementary Material

Supplemental Material

## Data Availability

All the data had been included in the manuscript, and the source files could be obtained from the corresponding author upon reasonable request.
