# Supplementary material for: Bone mesenchymal stem cells-derived miR-223-3p-containing exosomes ameliorate lipopolysaccharide-induced acute uterine injury via interacting with endothelial progenitor cells
Source: Bioengineered. 2021 Dec 7;12(2):10654–65. doi: 10.1080/21655979.2021.2001185 (PMC8810142; doi:10.1080/21655979.2021.2001185)
Supplement: Supplemental Material [file KBIE_A_2001185_SM6367.zip › supplementary/Supplementary Figure legends.docx]

**Supplementary Figure legends**

Figure S1. The ratio of Bax/Bcl-2 in mice tissues were determined by performing Real-Time qPCR analysis. Individual experiment repeated 3 times, and **P* < 0.05.
