# Supplementary figures and images for: Bone mesenchymal stem cells-derived miR-223-3p-containing exosomes ameliorate lipopolysaccharide-induced acute uterine injury via interacting with endothelial progenitor cells
Source: Bioengineered. 2021 Dec 7;12(2):10654–65. doi: 10.1080/21655979.2021.2001185 (PMC8810142; doi:10.1080/21655979.2021.2001185)

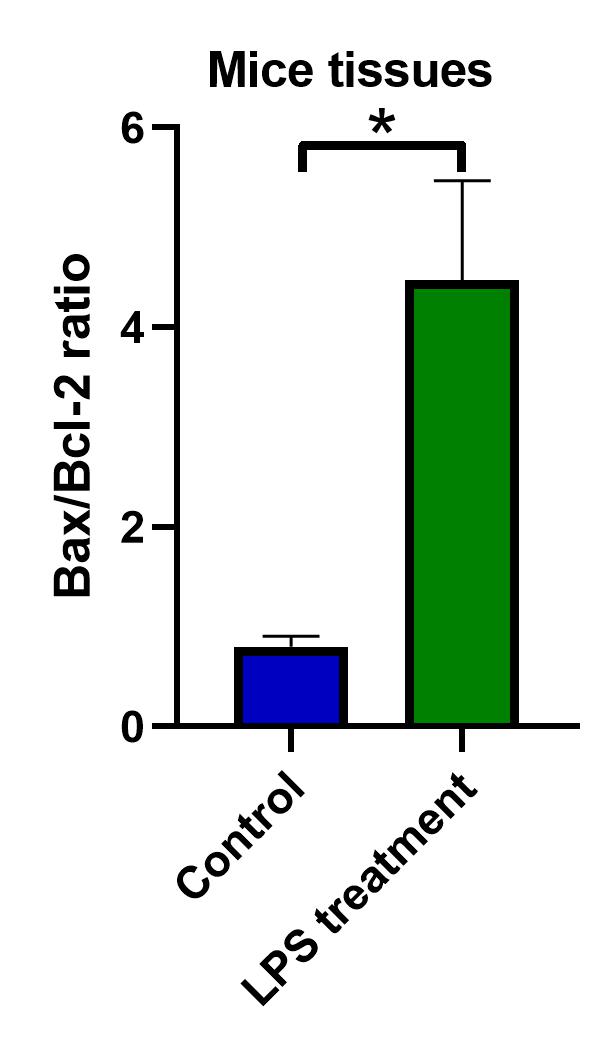

Supplement: Supplemental Material [file KBIE_A_2001185_SM6367.zip › supplementary/Figure S1.jpg]
